# Unravelling heterogeneity in efficiency measurements: a case study of public hospitals in Spain

**DOI:** 10.1007/s10198-025-01818-y

**Published:** 2025-07-30

**Authors:** Marta Arbelo-Pérez, Violeta De Vera, Antonio Arbelo

**Affiliations:** Grupo de Investigación EFIVENCOM, Departamento de Dirección de Empresas, Instituto Universitario de la Empresa (IUDE), Universidad de La Laguna (ULL), Campus de Guajara, San Cristóbal de La Laguna, Tenerife 38071 Spain

**Keywords:** Public healthcare, Hospital efficiency analysis, Stochastic frontier analysis (SFA), Bayesian methods, Hospital heterogeneity, D24, C11, H51, I14

## Abstract

Understanding efficiency in the hospital setting is now recognized as a fundamental pillar for making informed decisions and is crucial to ensuring the sustainability of health care systems. However, a significant portion of the literature assessing efficiency in the hospital sector tends to overlook heterogeneity across hospitals, leading to a notable bias in inefficiency estimation. In this paper, a stochastic frontier Bayesian model with random coefficients was used to estimate cost efficiency in the hospital sector, assuming heterogeneity across hospitals. The sample included 278 public hospitals in Spain during the period 2016–2019. The results revealed that public hospitals in Spain operate at a medium level of cost inefficiency of 12.86%, with this inefficiency being overestimated by 9.2% points if heterogeneity across hospitals is not adequately considered. This result underscores the importance of incorporating heterogeneity across hospitals in the evaluation of hospital efficiency to obtain accurate and reliable estimates.

## Introduction

The hospital sector has long faced the dual challenge of improving efficiency while maintaining high-quality care in an increasingly complex healthcare environment. Amid technological advancements, growing service demands, rising healthcare expenditures, and persistent budgetary pressures, resource optimization has become a strategic imperative to ensure both sustainability and excellence in service delivery. In this context, public officials are urging hospital managers to improve cost management, necessitating the exploration of new ways to monitor and control hospital finances and focusing on the efficient use of public resources [1, 2]. As a result, improving the efficiency of the hospital sector is essential for addressing the critical problems faced by the health system in general [3]. Importantly, efficiency in health care should not compromise quality or patient safety. The goal is to efficiently deliver high-quality health care.

An abundance of literature has evaluated efficiency in the hospital sector [2–14]. These investigations have focused mainly on estimating technical efficiency or cost efficiency through frontier models, which have been among the most commonly used performance measures in the hospital sector during the last decade. Although cost efficiency is particularly relevant for public hospital management, efficiency itself is a multifaceted and evolving concept, with strategies for improvement varying depending on factors such as geographic location, hospital size, specialization, and internal organization.

One critical issue that remains insufficiently addressed in this literature is hospital heterogeneity, which can significantly bias efficiency estimates and lead to suboptimal policy decisions. Each hospital has its own specific internal and environmental characteristics [15]. For example, some hospitals are more complex and have greater expertise in caring for more complicated pathologies than do other hospitals. Additionally, hospitals vary by size and mission; some are university hospitals, and some serve populations with different socioeconomic conditions. These differences are especially relevant in Spain, where each autonomous community manages its hospitals and where substantial variations in resources and socioeconomic conditions are found. Therefore, it seems realistic to assume that hospitals have very different characteristics that make them highly heterogeneous.

Heterogeneity has been a general concern in the analysis of efficiency [4] and has been addressed via multiple approaches [16–18]. However, the models used in the literature to account for heterogeneity have important limitations [19]. When heterogeneity is not adequately modelled, variations in inputs or technologies across hospitals may be incorrectly attributed to inefficiency. In this context, unobserved heterogeneity can be confounded with inefficiency, leading to erroneous conclusions [3, 20]. In essence, unmeasured heterogeneity can be disguised as inefficiency [21], which can lead to the conclusion that a hospital in which a new technology is not adopted is more inefficient than is another hospital simply because of a technological difference and not because of poor management, or vice versa. Consequently, not adequately accounting for heterogeneity can lead to inaccurate results by overestimating the inefficiency of hospitals. This situation is particularly relevant in Spain, where diversity across hospitals is a fundamental characteristic.

The purpose of this paper is to address this gap by examining the effect of heterogeneity across hospitals on the estimation of cost efficiency in a sample of 278 public hospitals in Spain between 2016 and 2019 via the application of stochastic frontier analysis (SFA) with fixed vs. random coefficients. To do so, initially, we assume that all hospitals are homogeneous and share the same cost frontier. We subsequently eliminate this unrealistic and restrictive assumption to account for heterogeneity across hospitals. This modelling approach allows the cost frontier to vary across hospitals, explicitly capturing structural and institutional differences, such as hospital size, service complexity, geographical setting, and regional governance. The aim of this study is to adopt this methodology to generate more accurate and context-sensitive efficiency estimates that are aligned with the operational realities of the Spanish healthcare system. This alignment not only enhances the validity of the results but also strengthens their practical relevance for performance benchmarking, hospital management, and policy design.

This study makes two key contributions to the literature. First, it applies a heterogeneity-aware methodology that enables a clearer separation between true inefficiency and structural variation, as separating heterogeneity from true inefficiency is difficult but essential for accurately understanding efficiency. Second, it provides empirical evidence on the extent and impact of hospital heterogeneity in Spain. The results obtained have the potential to offer a more precise and applicable vision for decision-making in hospital management and the formulation of health policies.

## Literature review

Efficiency and cost reduction are the quality parameters on which public hospitals are evaluated, and guaranteeing the sustainability of health care systems is essential [2, 22–25].

The literature on hospital efficiency is extensive and focuses primarily on the following two dimensions: technical efficiency [5, 6, 11, 26–30] and cost efficiency [9, 31–36]. Technical efficiency assesses the ability of a hospital to maximize its production with a given set of resources, whereas cost efficiency delves further by considering the cost implications of these resources [37, 38]. Although cost efficiency provides a more comprehensive view of hospital performance, obtaining accurate cost data poses challenges, leading many studies to concentrate on technical efficiency [20].

However, a critical aspect often overlooked in efficiency studies, both technical and cost related, is heterogeneity across hospitals. Most of the related literature relies on frontier models, which assume a common production possibility frontier and identical conditions for all hospitals. This approach can risk overestimating inefficiency, as different realities are not adequately considered [21, 39, 40]. Moreover, recent reviews such as Pai et al. [41] confirm that conventional data envelopment analysis (DEA) models remain dominant in the literature, with limited consideration given to hospital-level heterogeneity and context-specific modelling approaches.

Heterogeneity across hospitals presents many challenges; for example, it affects the process through which the state finances them. If hospitals are heterogeneous, then it is difficult to distinguish how much of the variation in expenses is attributed to effective cost containment measures and how much is due to unobservable hospital heterogeneity factors [42]. Therefore, properly recognizing and measuring heterogeneity are essential for rigorous efficiency analysis, as not considering heterogeneity can result in erroneous measurements. Recent studies have further emphasized how institutional frameworks and healthcare governance affect efficiency and heterogeneity across hospitals [36].

Numerous studies have explored the external and internal factors influencing hospital performance [38]. External factors include the environment’s impact on hospital finances, funding methods, and performance management [21, 43]. Internally, managerial practices, collaboration between doctors and managers, operational processes, and the use of personnel and technology affect efficiency [44, 45].

Consequently, various factors may affect the efficiency of each hospital, such as the type of property (public or private), whether it is a university hospital, the degree of specialty, the size and type of management, and the socioeconomic situation of the region it serves and the market in which it exists [46–53].

In light of this reality, some studies have attempted to address efficiency by incorporating heterogeneity into the analysis [4, 16, 18, 42, 54–57]. For instance, Auteri et al. [4] estimated the technical efficiency of Italian hospitals while considering spatial heterogeneity. However, this approach, which focuses on spatial factors, may not sufficiently capture the multifaceted nature of hospital heterogeneity.

Arvelo-Martín et al. [16] attempted, albeit insufficiently, to address heterogeneity, aiming to quantify the bias in the productivity measures of Spanish public hospitals by considering cost heterogeneity. Although this study incorporated observable and unobservable heterogeneity, it retained a common frontier for all hospitals, limiting its ability to capture true individual differences. Similarly, Colombi et al. [55] proposed a similar approach for Italian hospitals, incorporating exogenous variables affecting inefficiency levels. While contributing to understanding heterogeneity, these approaches impose a common frontier for all hospitals, potentially oversimplifying the intricate nature of individual hospital differences.

Similarly, Dormot and Milcent [42] explored the sources of variability in hospital costs in France. To address hospital heterogeneity, they used an econometric approach with panel data and developed a model of error components. Like the other works described, the above authors contributed to efficiency analyses by identifying the transitory and permanent components of heterogeneity in hospital costs; however, their methods were not very flexible and did not adequately capture the individual differences of each hospital. Rosko et al. [57] examined the relationship between the profitability and efficiency of nonprofit hospitals in the US and investigated those factors that may be related to profitability, such as the size or occupancy rate of the hospital. To do this, these authors used a traditional stochastic frontier model, thus yielding interesting information; however, they also assumed that all hospitals operate under a common technology and that differences in efficiency are due to technical inefficiency and not to structural differences across hospitals. In Herr [56], a similar dynamic was observed; the objective was to identify how hospital efficiency varies with the type of hospital ownership and other exogenous factors. To do this, both technical efficiency and cost efficiency were calculated via traditional SFA.

A noteworthy contribution to the field is the analysis conducted by Andrews and Emvalomatis [54], who examined the cost efficiency of New Zealand District Health Boards (DHB) by applying a dynamic stochastic frontier model. This model incorporates random and DHB-specific effects, which facilitate the adjustment of both the observed and unobserved heterogeneity in the cost function. This particular methodology implies that each DHB possesses its own random effect within the model, whereas the efficiency frontier remains common across all entities.

To our knowledge, only three studies, all employing frontier models with random parameters, have adequately captured heterogeneity, focusing on hospitals in Switzerland [25, 58] and public hospitals in Tunisia [59]. The findings from the study conducted by Zweifel and Widmer [25] affirmed the presence of unobserved heterogeneity, and the consideration of this heterogeneity resulted in a 5% reduction in the estimated average inefficiency. Hence, their conclusion underscores the inadequacy of the conventional SFA model for a thorough examination. The above authors emphasized the significance of these findings since, in the current context in which many hospitals have extended prospective payments, hospitals that have good management but are burdened by obsolete technology can be penalized. Similarly, in the context of Tunisia, the study by Chaabouni and Abednnadher [59] reinforced the suitability of this methodology, demonstrating its alignment with the data and its capacity to mitigate biases by distinguishing the estimation of cost efficiency from that of technological disparities.

While earlier studies have contributed to efficiency analyses, their applicability is limited to specific contexts. Switzerland, New Zealand and Tunisia, chosen as study cases, present diverse realities across economic, cultural, demographic, and social dimensions. Consequently, the findings from these studies may not be directly extrapolated to other national contexts. Each country possesses unique challenges and strengths within the health care sector, which requires analyses and approaches adapted to its specific circumstances. This situation underscores the importance of examining Spain, a context of particular interest. Within the literature focused on Spanish public hospitals, the predominant focus has been on evaluating technical efficiency rather than cost efficiency. Commonly, DEA has been employed, with many studies concentrating on a specific autonomous community [23, 24, 60–64].

In summary, although various investigations have attempted to determine the differences in resources and socioeconomic conditions under which hospitals operate, the problem of heterogeneity across hospitals has not been satisfactorily solved. This study aims to contribute to the literature by evaluating changes in the measurement of the inefficiency of public hospitals in Spain when heterogeneity is appropriately taken into account.

## Methodology

The evaluation of cost efficiency typically relies on parametric methods, such as SFA, or nonparametric approaches, such as DEA. Introduced by Charnes, Cooper, and Rhodes [65], DEA uses linear programming techniques to establish an efficiency frontier. In the realm of hospitals, this method has been applied by various authors, including Banker, Conrad, & Strauss [66]; Fizel and Nunnikhoven [67]; Kooreman [68], Parkin and Hollingsworth [69]; Burgess and Wilson [70]; Rollins, Lee, Xu, & Ozcan [71]; Steinmann and Zweifel [72]; and Steinmann, Dittrich, Karmann, and Zweifel [73].

However, while DEA is generally easier to estimate [17], it overlooks pricing factors and, consequently, can measure only technical efficiency. Another limitation is that it considers any deviation between a hospital and the efficient frontier as inefficiency, disregarding the potential influence of random errors. Moreover, the location of the efficiency frontier may depend on an extreme unit in the input or output space due to the absence of a comparator.

SFA, conversely, is a methodology that employs econometric methods to estimate efficiency. Simultaneously introduced by Aigner, Lovell, and Schmidt [74] and Meeusen and Van den Broeck [75], SFA is a parametric technique that aligns better with the previously discussed concept of cost efficiency. In SFA, the cost of a specific hospital is assessed in relation to the efficiency frontier. Any deviation from this frontier is attributed to both inherent inefficiency and random errors resulting from data collection failures, as well as potential unobservable variables beyond hospital control [76].

Although both parametric and nonparametric methods have certain merits and drawbacks, our analysis adopts SFA. This choice is driven by the ability of SFA to capture stochastic factors and measurement errors, which are highly relevant in the health care sector. In contrast to nonparametric methods such as DEA, which do not account for random errors and attribute all deviations from the frontier to inefficiency, SFA allows for a more refined decomposition of the error term. Given the nature of our data and research objectives, we consider the SFA framework more appropriate. The SFA approach has been extensively utilised in the study of hospital efficiency [10, 16, 46, 58, 77, 78].

Traditionally, cost efficiencies have been estimated using the SFA methodology under the assumption of fixed model parameters, which implies that parameters remain constant across hospitals, assuming homogeneity in both internal and external factors. The SFA model with fixed coefficients for cost efficiency estimation can be expressed as follows:

1 $$\:{C}_{i,t}=f({x}_{i,t},{w}_{i,t},\beta\:,\:\delta\:)\cdot\:\mathrm{e}\mathrm{x}\mathrm{p}({u}_{i,t}+{v}_{i,t})$$

where.

- $\:f$ (⋅) is a deterministic function that describes the relationships among outputs, input prices and costs.
- $\:{x}_{i,t}$ is the vector of outputs for hospital $\:i$ in year $\:t$.
- $\:{w}_{i,t}$is the vector of input prices for hospital $\:i$ in year $\:t$.
- $\:\beta\:$ and $\:\delta\:$ are vectors of fixed coefficients.
- $\:{u}_{i,t}$ is the inefficiency term.
- $\:{v}_{i,t}$ is the error term capturing the stochastic variability in costs.

However, the reality is that the resources and the organizational structure of Spanish hospitals for providing health care services are inherently heterogeneous. As previously mentioned, variations in size, location, complexity, and specialization across hospitals exist. Compared with smaller or general hospitals, larger or specialized hospitals exhibit distinct structures and processes. Clinical protocols and standard operating procedures also vary, impacting the efficiency of health care service delivery. Additionally, geographical location influences the demand for health services and the availability of resources and medical personnel. The composition and needs of the served population vary significantly, with hospitals serving older people facing different challenges than are those catering to younger people.

Hence, assuming homogeneity across hospitals introduces bias and can lead to errors in efficiency estimation, complicating direct comparisons across hospitals. Individual differences may render the efficiency of one hospital incomparable to that of another hospital, as these differences significantly influence efficiency outcomes.

To address this issue, we propose the utilization of the SFA model with random coefficients, as suggested by Tsionas [40]. This model extends traditional SFA by allowing model parameters to vary randomly among the studied hospitals. Random coefficients accommodate specific heterogeneity in each unit, as different hospitals may have distinct production technologies or cost structures. Moreover, random coefficients can be employed to account for unobserved factors that systematically influence the production process but are not explicitly included in the model.

The SFA model with random coefficients for cost efficiency estimation can be expressed as follows:

2 $$\:{C}_{i,t}=f({x}_{i,t},{w}_{i,t},{\beta\:}_{i},\:{\delta\:}_{i})\cdot\:\mathrm{e}\mathrm{x}\mathrm{p}({u}_{i,t}+{v}_{i,t})$$

where

- $\:{\beta\:}_{i}$ and$\:\:{\delta\:}_{i}$ are the vectors of coefficients that vary randomly across hospitals.

To reveal the genuine significance of distinctions across hospitals, we initially presume homogeneity across these institutions, positing that they share an identical cost frontier. Specifically, Eq. (1) is employed for estimation. Subsequently, we proceed to integrate heterogeneity by introducing random coefficients into the model, as denoted in Eq. (2). By incorporating random coefficients, the approach accounts for hospital-specific variation in the cost frontier, capturing institutional characteristics such as size, the complexity of services, location, and regional governance. As a result, it yields more accurate and context-sensitive efficiency estimates that reflect the structural and operational diversity of the Spanish healthcare system.

## Data and empirical model

### Data

The data used in this study were obtained from the Statistics of Specialized Care Health Centers (SIAE) provided by the Spanish Ministry of Health. Microdata extracted from surveys conducted between 2016 and 2019 formed the basis of our analysis. The database offers comprehensive information on Spanish hospitals, with a specific focus on public acute hospitals. Autonomous communities that were statistically grouped with other geographic areas or lacked complete information for all study years were excluded. The variables considered in this database were hospital personnel, outpatient consultations, hospital discharge, emergencies, surgical interventions, beds, personnel costs, provisions for amortization, investments and purchases.

The period 2016–2019 was deliberately selected to capture hospital performance under stable, pre-pandemic conditions. The COVID-19 pandemic, which began in 2020, introduced significant and atypical disruptions to hospital activity across Spain, including shifts in demand patterns, emergency restructuring of services, changes in resource allocation, and variations in cost structures. These factors introduced atypical and exogenous variations that do not reflect structural inefficiency. Therefore, including pandemic-era data could distort the estimation of hospital efficiency. Focusing on the pre-pandemic period ensures consistency and comparability across hospitals and provides a clearer picture of performance under routine operating conditions.

In addition, we used data from the Registry of Specialized Care Activity– Basic Minimum Data Set (RAE-CMBD) of system hospitals, also provided by the Spanish Ministry of Health. This database provides information on the average Spanish case-mix adjustment, which is calculated as the weighted average of diagnosis-related group (DRG) weights. Each DRG was assigned a weight reflecting the complexity and resources required for patient treatment. Hospital discharge data were adjusted by multiplying them by this case-mix adjustment variable. Finally, the Health Barometer survey, a collaborative effort between the Spanish Ministry of Health and the Sociological Research Center (CIS), contributed information on patient satisfaction, offering insights into the patient experience.

The selection of input and output variables in this study is grounded in established practices in the hospital efficiency literature. As outputs, we include weighted hospital discharges (adjusted by the DRG case mix), outpatient consultations, emergency visits, surgical interventions, and patient satisfaction. Weighted discharges are commonly used to control for heterogeneity in patient complexity and have been recommended to ensure comparability across hospitals with different case profiles [55, 63, 79]. Outpatient, emergency, and surgical activities are widely accepted as standard indicators of hospital production [37, 60]. Although patient satisfaction is less frequently used, it has been incorporated as a proxy for perceived quality in several studies [24, 38], in line with the growing emphasis on value-based healthcare.

Regarding inputs, we consider the prices of labour, capital, and materials. These are operationalized as unit costs derived from hospital expenditure data, a method that is consistent with previous research using both parametric and non-parametric frontier models [56, 57, 80]. The labour cost per employee, capital cost based on depreciation, and material costs per unit of input are commonly employed proxies to capture the economic resources consumed in hospital production [58, 60]. Overall, variable selection is consistent with methodological precedents in the hospital efficiency literature in Spain and internationally.

The descriptive statistics for the variables used are displayed in Table 1. To mitigate the impact of price fluctuations, monetary variables are adjusted using the general consumer price index, with the base year set as 2021.

**Table 1:** Overview of descriptive statistics

Variable	Symbol	Mean	SD	Minimum	Maximum
Costs ^*^	$\:C$	138,776.69	146,342.19	2,175.05	764,742.38
Weighted discharges	$\:{x}_{1}$	13,317.88	12,069.50	107.91	57,092.19
External consultations	$\:{x}_{2}$	267,501.95	239,723.45	1,199	1,358,274
Emergencies	$\:{x}_{3}$	76,574.57	61,087.84	26	398,121
Surgical interventions	$\:{x}_{4}$	12,079.46	10,811.56	107	58,203
Satisfaction	$\:{x}_{5}$	6.64	0.34	6.00	7.45
Price of materials	$\:{w}_{1}$	166,241.38	66,460.98	20,889.75	412,664.68
Price of capital	$\:{w}_{2}$	5.850,74	8,205.62	0	50,968.33
Price of labour	$\:{w}_{3}$	55.855,47	9,883.40	17,186.77	96,636.40

^*^ in thousands of euros

### Empirical model

In the realm of efficiency estimation, the choice of functional form is a pivotal step in the SFA modelling process. The translog functional form has emerged as a frequently employed structure because of its flexibility in modelling the relationships among outputs, input prices, and costs. This flexibility is particularly valuable in relaxing constraints on the constant elasticity of substitution.

The translog function within the SFA model, characterized by fixed coefficients as per Eq. (1) and imposing linear homogeneity in inputs, can be expressed as follows:

3 $$\eqalign{ ln({{\>{C_{it}}} \over {{w_{3,it}}}}) & = \beta {\>_0} + \sum {\>_{j = 1}^5} \beta {\>_j}ln{x_{j,it}} + \sum {\>_{s = 1}^2} \delta {\>_s}ln({{{w_{s,it}}} \over {{w_{3,it}}}}) \cr & + + {1 \over 2}\sum {\>_{j = 1}^5} \sum {\>_{k = 1}^5} \beta {\>_{jk}}ln{x_{j,\>it}}ln{x_{k,it}} \cr & + {1 \over 2}\sum {\>_{s = 1}^2} \sum {\>_{r = 1}^2} \delta {\>_{sr}}ln({{{w_{s,\>it}}} \over {{w_{3,it}}}})ln\left( {{{{w_{r,it}}} \over {{w_{3,it}}}}} \right) \cr & + + \sum {\>_{j = 1}^5} \sum {\>_{s = 1}^2} \rho {\>_{js}}ln{x_{j,\>it}}{\rm{ln}}({{{w_{s,it}}} \over {{w_{3,it}}}}) + {v_{it}} + {u_{it}} \cr} $$

where

- $\:{C}_{it}$ is the total cost for hospital $\:i$ in year $\:t$.
- $\:{x}_{i,t}$ is the vector of outputs and is selected in this study as follows:
  - $$\:{x}_{1}$$ =$$\:Weighted\:discharges$$
  - $$\:{x}_{2}$$ =$$\:External\:consultations$$
  - $$\:{x}_{3}$$ = *Emergencies*.
  - $$\:{x}_{4}$$ =$$\:Surgical\:interventions$$
  - $$\:{x}_{5}$$ =$$\:Satisfaction$$
- $\:{w}_{i,t}$ is the vector of input prices and is selected in this study as follows:
  - $$\:{w}_{1}$$ =$$\:Price\:of\:materials$$
  - $$\:{w}_{2}$$ =$$\:Price\:of\:capital$$
  - $$\:{w}_{3}$$ =$$\:Price\:of\:labour$$
- $\:{\beta\:}_{0}$ is the intercept.

Furthermore, the translog function within the SFA model, characterized by random coefficients as described by Eq. (2) and enforcing linear homogeneity in inputs, can be expressed as follows:

4 $$\eqalign{ ln({{\>{C_{it}}} \over {{w_{3,it}}}}) & = \beta {\>_0} + \sum {\>_{j = 1}^5} \beta {\>_{j,i}}ln{x_{j,it}} + \sum {\>_{s = 1}^2} \delta {\>_{s,i}}ln({{{w_{s,it}}} \over {{w_{3,it}}}}) \cr & + + {1 \over 2}\sum {\>_{j = 1}^5} \sum {\>_{k = 1}^5} \beta {\>_{jk,i}}ln{x_{j,\>it}}ln{x_{k,it}} \cr & + {1 \over 2}\sum {\>_{s = 1}^2} \sum {\>_{r = 1}^2} \delta {\>_{sr,i}}ln({{{w_{s,\>it}}} \over {{w_{3,it}}}})ln\left( {{{{w_{r,it}}} \over {{w_{3,it}}}}} \right) \cr & + + \sum {\>_{j = 1}^5} \sum {\>_{s = 1}^2} \rho {\>_{js,i}}ln{x_{j,\>it}}{\rm{ln}}({{{w_{s,it}}} \over {{w_{3,it}}}}) + {v_{it}} + {u_{it}} \cr} $$

For the estimation of Models (3) and (4), Bayesian inference is chosen as the statistical approach. This approach involves updating beliefs about unknown parameters as new evidence or data are incorporated. Unlike frequentist inference, which considers parameters as fixed and data as random variables, Bayesian inference treats parameters as random variables and uses the prior distribution, likelihood, and subsequent distribution to quantify the uncertainty of these parameters.

The steps followed in Bayesian inference are as follows:

1. Before the data are observed, the prior distributions of the parameters are specified. In our case, the following distributions are imposed on the parameters to be estimated:
  - $\:{\beta\:}_{0}$ follows a normal distribution with a mean of zero and a variance of $\:{10}^{-6}$, such that$\:{\:\beta\:}_{0}\:\sim\:N(0,{10}^{-6})$.
  - $\:{\beta\:}_{i}$, $\:{\delta\:}_{i}$, and $\:{\rho\:}_{i}$ follow a multivariate normal distribution with means of $\:\stackrel{-}{\beta\:}$, and $\:\stackrel{-}{\delta\:},\:\stackrel{-}{\rho\:}$ and a variance of $\:\varOmega\:$, where $\:\varOmega\:$ is an inverted Wishart distribution. In turn, $\:\stackrel{-}{\beta\:}$ and $\:\stackrel{-}{\delta\:},\:\stackrel{-}{\rho\:}$ follow a normal distribution with a mean of zero and a variance of $\:{10}^{-6}$ such that $\:\stackrel{-}{\beta\:}$ and $\:\stackrel{-}{\delta\:},\:\stackrel{-}{\rho\:\:}\sim\:N(0,{10}^{-6})$.
  - $\:{u}_{i,t}$ follows a truncated normal distribution such that $\:{u}_{i,t}\:\sim{N}^{+}(0,\:{\sigma\:}_{u}^{2})$ and$\:\:{\sigma\:}_{u}^{2}\:\sim$ G$\:(5,{\gamma\:}_{0})$, where $\:{\gamma\:}_{0}$ = 5 * ln ($\:{r}^{*}$) * ln ($\:{r}^{*}$). The parameter$\:\:{r}^{*}$ represents the prior mean efficiency value.
  - $\:{v}_{it}$ follows a normal distribution with a mean of zero and a variance of τ, where τ is the precision parameter (inverse of variance). In turn, τ follows a gamma distribution such that $\:{\uptau\:}\:\sim\:G\left(\mathrm{0.001,0.001}\right)$.

2) The relationship between the observed data and parameters is modelled through the likelihood.
3) After the data are observed, the posterior distribution is calculated using Bayes’ theorem. The posterior distribution combines prior information and likelihood information to provide the updated distribution of the parameters given the observed data.

## Results

This study assesses the cost efficiency of a representative sample of 278 Spanish public hospitals over the period 2016–2019, employing stochastic frontier models that explicitly account for structural heterogeneity within the Spanish healthcare system. The estimation of Eqs. (3) and (4) is conducted via WinBUGS 14. The results, reported in Tables 2 and 3, present the posterior means and 97.5% credibility intervals for the parameters defining the cost frontier with fixed coefficients (assuming homogeneity) and random coefficients (assuming heterogeneity), respectively. These estimates are derived via Markov chain Monte Carlo (MCMC) simulation comprising 100,000 iterations, with the initial 20,000 iterations discarded to mitigate any sensitivity to initial values.

The fixed coefficient model, in which all hospitals are assumed to operate under a common production frontier (Eq. 3), yields an average efficiency level of 77.94% (see Table 4). This result suggests that, under the homogeneity assumption, Spanish public hospitals could reduce their total costs by approximately 22.06% without compromising the level of assistance. This finding aligns with earlier European studies in which unobserved heterogeneity often leads to an upward bias in inefficiency estimates [25, 58].

To capture the structural and operational diversity characteristics of decentralized European healthcare systems, particularly those with regional governance, such as Spain, we estimate a random coefficient model (Eq. (4)). This model allows hospital-specific variations in the cost frontier, reflecting institutional factors such as geographical disparities, service complexity, and hospital size. The average efficiency in this model increases significantly to 87.14%, implying a reduced inefficiency margin of 12.86% and highlighting the importance of controlling for latent heterogeneity. This improvement in accuracy demonstrates that the failure to account for heterogeneity can lead to a systematic overestimation of inefficiency.

**Fig. 1:**
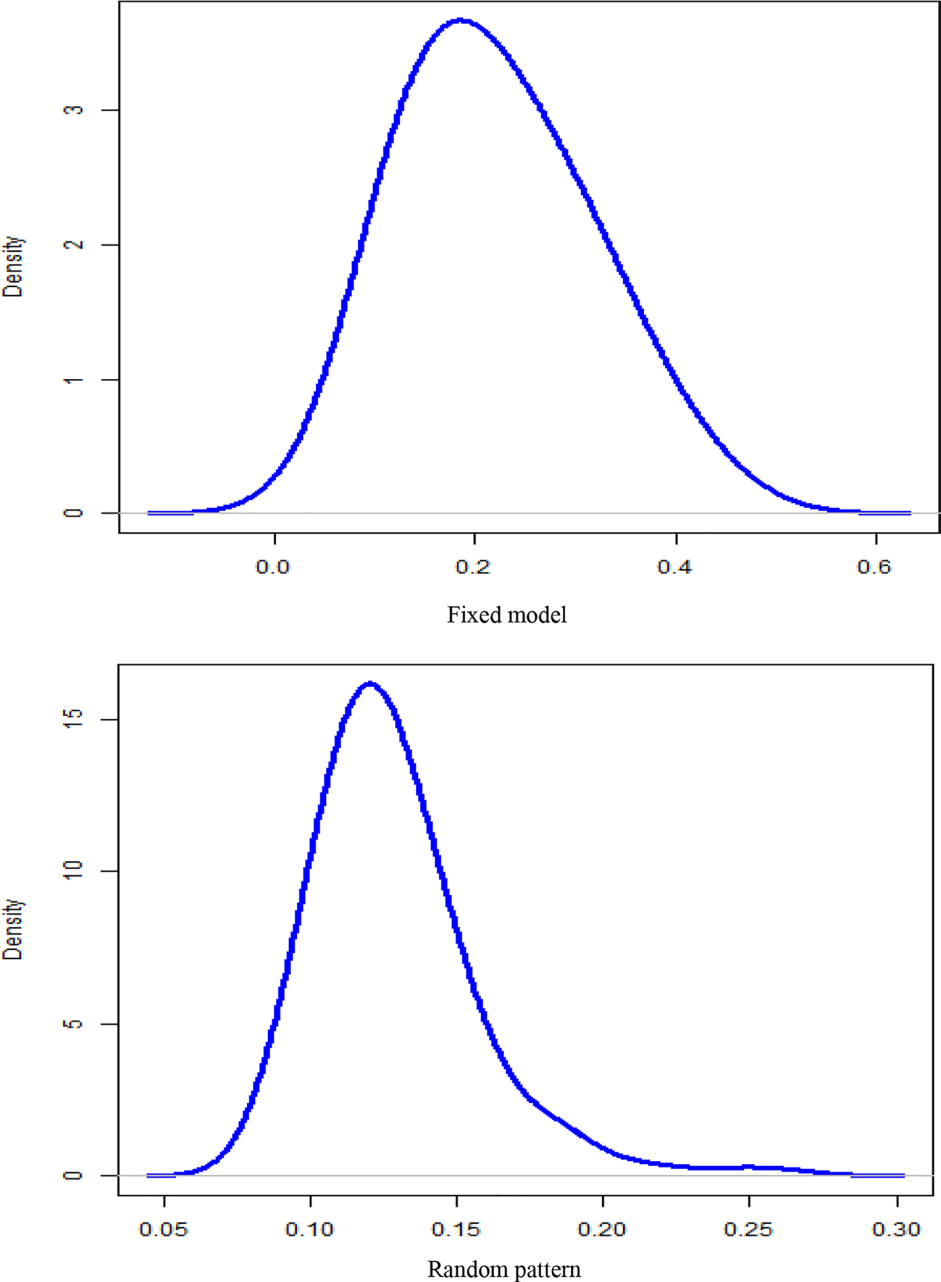
Distribution of the probability of cost inefficiency

The improved efficiency scores under the random coefficient specification not only enhance the reliability of the estimates but also reflect the institutional realities of hospital management within the Spanish National Health System. These results are visualized in Fig. 1, which compares the posterior distributions of inefficiency across models. In the fixed coefficient setting, the inefficiency distribution peaks at approximately 0.2, whereas in the random coefficient model, this distribution peaks at approximately 0.11, indicating significantly higher efficiency when hospital-level heterogeneity is properly modelled. The narrower spread in the latter distribution further suggests reduced variability in inefficiency estimates across hospitals. This reduced variability may reflect greater consistency in performance assessment and could contribute to more equitable resource allocation strategies.

Overall, these findings underscore the importance of heterogeneity-adjusted modelling frameworks in European health economics research, especially in settings where hospitals operate under diverse policy, demographic, and geographic conditions. Such approaches may also be adaptable to other decentralized health systems across Europe, such as those of Germany or Italy, where regional variation is similarly relevant. The results have direct implications for benchmarking, resource allocation, and the design of hospital performance incentives within multi-tiered healthcare systems.

**Table 2:** Bayesian cost frontier parameter estimation (fixed coefficients)

Parameter	Mean	97.5% interval	Parameter	Mean	97.5% interval
$\:{\beta\:}_{0}$	15.200	[15.04, 15.33]	$\:{\stackrel{-}{\beta\:}}_{34}$	-0.008	[-0.02, 0.0002]
$\:{\stackrel{-}{\beta\:}}_{1}$	-0.927	[-0.97, -0.89]	$\:{\stackrel{-}{\beta\:}}_{35}$	0.191	[0.17, 0.22]
$\:{\stackrel{-}{\beta\:}}_{2}$	-0.544	[-0.56, -0.52]	$\:{\stackrel{-}{\beta\:}}_{44}$	0.179	[0.17, 0.19]
$\:{\stackrel{-}{\beta\:}}_{3}$	-0.802	[-0.82, -0.78]	$\:{\stackrel{-}{\beta\:}}_{45}$	0.245	[0.21, 0.28]
$\:{\stackrel{-}{\beta\:}}_{4}$	-0.380	[-0.40, -0.35]	$\:{\stackrel{-}{\beta\:}}_{55}$	0.019	[-0.002, 0.04]
$\:{\stackrel{-}{\beta\:}}_{5}$	0.273	[0.26, 0.28]	$\:{\stackrel{-}{\delta\:}}_{11}$	0.106	[0.09, 0.12]
$\:{\stackrel{-}{\delta\:}}_{1}$	-0.438	[-0.47, -0.41]	$\:{\stackrel{-}{\delta\:}}_{12}$	0.001	[-0.01, 0.01]
$\:{\stackrel{-}{\delta\:}}_{2}$	-0.062	[-0.09, -0.03]	$\:{\stackrel{-}{\delta\:}}_{22}$	0.010	[-0.001, 0.02]
$\:{\stackrel{-}{\beta\:}}_{11}$	0.188	[0.16, 0.23]	$\:{\stackrel{-}{\rho\:}}_{11}$	-0.195	[-0.21, -0.18]
$\:{\stackrel{-}{\beta\:}}_{12}$	0.187	[0.15, 0.22]	$\:{\stackrel{-}{\rho\:}}_{12}$	0.087	[0.07, 0.10]
$\:{\stackrel{-}{\beta\:}}_{13}$	-0.011	[-0.03, 0.01]	$\:{\stackrel{-}{\rho\:}}_{21}$	0.528	[0.51, 0.55]
$\:{\stackrel{-}{\beta\:}}_{14}$	-0.369	[-0.40, -0.34]	$\:{\stackrel{-}{\rho\:}}_{22}$	-0.196	[-0.21, -0.18]
$\:{\stackrel{-}{\beta\:}}_{15}$	0.468	[0.4456, 0.49]	$\:{\stackrel{-}{\rho\:}}_{31}$	-0.258	[-0.28, -0.24]
$\:{\stackrel{-}{\beta\:}}_{22}$	-0.048	[-0.07, -0.02761]	$\:{\stackrel{-}{\rho\:}}_{32}$	0.098	[0.09, 0.10]
$\:{\stackrel{-}{\beta\:}}_{23}$	0.031	[0.01, 0.06]	$\:{\stackrel{-}{\rho\:}}_{41}$	0.086	[0.08, 0.10]
$\:{\stackrel{-}{\beta\:}}_{24}$	0.152	[0.14, 0.17]	$\:{\stackrel{-}{\rho\:}}_{42}$	0.028	[-0.01, 0.06]
$\:{\stackrel{-}{\beta\:}}_{25}$	-0.846	[-0.86, -0.83]	$\:{\stackrel{-}{\rho\:}}_{51}$	-0.769	[-0.79, -0.75]
$\:{\stackrel{-}{\beta\:}}_{33}$	0.012	[-0.01, 0.03]	$\:{\stackrel{-}{\rho\:}}_{52}$	0.193	[0.17, 0.21]

**Table 3:** Bayesian cost frontier parameter Estimation (random coefficients)

Parameter	Mean	97.5% interval	Parameter	Mean	97.5% interval
$\:{\beta\:}_{0}$	38.230	[11.12, 64.92]	$\:{\beta\:}_{34}$	0.060	[-0.002, 0.12]
$\:{\beta\:}_{1}$	-0.002	[-2.39, 2.41]	$\:{\beta\:}_{35}$	1.472	[0.82, 2.13]
$\:{\beta\:}_{2}$	1.705	[-0.24, 3.66]	$\:{\beta\:}_{44}$	0.269	[0.08, 0.45]
$\:{\beta\:}_{3}$	-1.868	[-3.11, -0.64]	$\:{\beta\:}_{45}$	0.258	[-0.85, 1.38]
$\:{\beta\:}_{4}$	-0.391	[-2.54, 1.76]	$\:{\beta\:}_{55}$	21.460	[8.32, 34.44]
$\:{\beta\:}_{5}$	-38.260	[-64.47, -11.68]	$\:{\delta\:}_{11}$	-0.443	[-0.62, -0.27]
$\:{\delta\:}_{1}$	-1.053	[-3.03, 0.92]	$\:{\delta\:}_{12}$	-0.022	[-0.06, 0.01]
$\:{\delta\:}_{2}$	0.314	[-0.14, 0.77]	$\:{\delta\:}_{22}$	0.017	[-0.0008,0.03]
$\:{\beta\:}_{11}$	0.317	[0.20, 0.43]	$\:{\rho\:}_{11}$	-0.181	[-0.32, -0.04]
$\:{\beta\:}_{12}$	-0.078	[-0.21, 0.05]	$\:{\rho\:}_{12}$	0.042	[0.007,0.08]
$\:{\beta\:}_{13}$	0.020	[-0.05, 0.09]	$\:{\rho\:}_{21}$	0.191	[0.07, 0.31]
$\:{\beta\:}_{14}$	-0.164	[-0.29, -0.04]	$\:{\rho\:}_{22}$	-0.075	[-0.12, -0.03]
$\:{\beta\:}_{15}$	-0.087	[-1.31, 1.13]	$\:{\rho\:}_{31}$	-0.128	[-0.21, -0.05]
$\:{\beta\:}_{22}$	0.494	[0.39, 0.59]	$\:{\rho\:}_{32}$	-0.011	[-0.04, 0.02]
$\:{\beta\:}_{23}$	-0.199	[-0.26, -0.14]	$\:{\rho\:}_{41}$	-0.035	[-0.18, 0.11]
$\:{\beta\:}_{24}$	-0.148	[-0.30, 0.01]	$\:{\rho\:}_{42}$	0.030	[-0.01, 0.06]
$\:{\beta\:}_{25}$	-1.530	[-2.54, -0.52]	$\:{\rho\:}_{51}$	1.190	[0.20, 2.19]
$\:{\beta\:}_{33}$	0.060	[0.04, 0.08]	$\:{\rho\:}_{52}$	0.056	[-0.16, 0.27]

**Table 4:** Estimation of cost efficiency (%)

	2016	2017	2018	2019	Mean
**Cost efficiency** (fixed coefficients)	77.42	78.69	78.21	77.47	**77.94**
**Cost efficiency** (random coefficients)	87.80	88.08	87.15	85.53	**87.14**

## Discussion

The findings of this study provide robust evidence on the cost efficiency of Spanish public hospitals, and they meaningfully contribute to the broader European debate on performance assessment in public healthcare systems. By contrasting fixed coefficient models with those incorporating random coefficients, our analysis highlights the importance of accounting for hospital-level heterogeneity, an approach that is increasingly being endorsed in European health economics. Standard models based on homogeneity often fall short in capturing the operational complexity of hospitals, particularly within decentralized systems such as those of Spain, where structural and institutional diversity is inherent.

The increase in observed efficiency from 77.94% under the fixed coefficient model to 87.14% under the random coefficient model reflects a substantial improvement in measurement precision. This 9.2% point reduction in estimated inefficiency, once heterogeneity is properly accounted for, reinforces a key insight in the European efficiency literature: overlooking institutional and contextual diversity leads to systematically biased results, potentially distorting funding allocations and performance-based policies. Similar findings in Switzerland obtained by Widmer et al. [58] and Zweifel and Widmer [25] corroborate this point, although the bias observed in those studies is slightly smaller (6% points), likely due to a more homogeneous hospital structure.

From a policy perspective, the implications are significant. The random coefficient approach yields a more accurate and context-sensitive evaluation of hospital performance, which is essential for informing national and regional health authorities within the EU seeking to optimize spending under constrained public budgets. In Spain, the average cost of €138.8 million per hospital suggests a potential efficiency gain of €17.85 million per hospital, funds that could be redirected to other pressing healthcare needs or improved service delivery.

These findings highlight the necessity of moving beyond uniform policies. Health systems across Europe differ in terms of governance, financing, population health needs, and organizational complexity. Hence, analytical tools and performance frameworks must reflect this diversity. A uniform application of benchmarking tools risks penalizing hospitals that operate under more complex conditions or, conversely, masking inefficiencies in better resourced institutions.

Our results emphasize that heterogeneity-aware models not only improve the statistical validity of efficiency scores but also facilitate better targeted managerial interventions. In the Spanish context, this could translate into differentiated strategies such as restructuring care pathways in large urban hospitals or optimizing resource use in smaller rural facilities.

Moreover, this study adds to the call within European health economics to adopt more sophisticated frontier models in comparative efficiency analyses. Policymakers at both the national level and the EU level are encouraged to promote flexibility in performance evaluation tools and to invest in data systems that allow for hospital-level customization. Doing so is particularly important in multi-level governance settings, where regional health authorities may hold autonomy over implementation but rely on centrally defined efficiency criteria.

At the managerial level, hospital administrators should incorporate heterogeneity-sensitive benchmarking tools to identify specific inefficiencies and redesign processes accordingly. The adoption of such tools can lead not only to cost containment but also to improved service quality and patient outcomes, aligning with broader EU objectives with regard to health system sustainability and resilience.

In conclusion, the integration of hospital-specific characteristics into efficiency measurement models is essential for generating reliable information to guide both operational decision-making and strategic policy formulation. This is a prerequisite for achieving equitable and efficient healthcare systems across Europe.

## Conclusions

Accurately assessing the cost efficiency of public hospitals is essential for sustaining health systems across Europe, particularly within decentralized frameworks in which institutions operate under diverse structural and contextual conditions. This study provides robust empirical evidence on the efficiency of 278 Spanish public hospitals between 2016 and 2019, highlighting the critical role of hospital-level heterogeneity in health economics modelling. Several key conclusions can be drawn:

1. Adjusting for heterogeneity enhances the validity of efficiency estimates. Models that incorporate random coefficients to account for hospital-specific differences report significantly higher average efficiency (87.14%) than do models that assume homogeneity (77.94%). The 9.2 percentage point discrepancy confirms that neglecting heterogeneity leads to a systematic overestimation of inefficiency, an issue with direct implications for performance-based funding and benchmarking practices common in European health systems.
2. Stochastic frontier models with random coefficients generate more policy-relevant and operationally useful insights. The stochastic specification separates true inefficiency from random variation caused by measurement error, reporting inconsistencies, or external shocks, all of which are common in administrative hospital data. Simultaneously, the inclusion of random coefficients allows for a more accurate representation of institutional diversity in terms of size, service complexity, geographic location, and governance structures. As a result, both local administrators and national health authorities are better equipped to design targeted, evidence-based interventions. In the context of EU priorities for value-based healthcare and data-driven governance, such methods are essential tools for improving accountability and resource management.
3. There is significant untapped potential for cost containment. Spanish public hospitals could reduce their costs by an average of 12.86% without compromising the level of assistance, which is equivalent to potential annual savings of approximately €17.85 million per hospital. For health systems under fiscal pressure, this finding underscores the importance of efficiency analysis as a tool for optimizing expenditures without reducing access or quality.
4. The findings are consistent with those of the international literature while highlighting the structural specificity of Spain. Similar studies in Switzerland have shown efficiency overestimations when heterogeneity is not addressed, but the larger effect observed in Spain suggests greater institutional diversity. This reinforces the need to adapt cross-country comparative models and EU-level efficiency assessments to reflect national and regional particularities rather than applying uniform methodologies.
5. Policy design must recognize the multi-level governance of European health systems. In countries with decentralized or federated health systems, such as Spain, Germany, or Italy, uniform policy prescriptions risk inefficiency and unfairness. The results advocate for flexible policy instruments that are responsive to hospital-level data and regional contexts, an approach that is increasingly aligned with EU health policy priorities such as subsidiarity and tailored care.

These conclusions have strategic implications for hospital management and health policy formulation. Integrating heterogeneity into efficiency models offers a more nuanced and realistic understanding of resource use, strengthening the foundation for performance-based reforms and contributing to a more resilient and sustainable public health sector. In decentralized systems such as those of Spain, this also demands coordination between national policy frameworks and regional implementation capacities.

However, the study is not without limitations. Its findings are context specific and dependent on the availability of administrative data. Future research should aim to replicate this approach across different European countries and health system configurations and to explore the application of this approach to other levels of care or integrated service delivery models. Furthermore, as the health sector evolves, due to demographic shifts and technological innovation, efficiency evaluation tools must also adapt.

In conclusion, this study contributes to the growing body of European health economics literature that calls for more context-sensitive, heterogeneity-aware approaches to measuring efficiency. Such methods are not merely academic improvements; they are essential instruments for managing public resources responsibly, guiding reform, and ensuring equitable and efficient healthcare delivery across diverse European health systems.
